# Design and implementation of a sexual health intervention for migrant construction workers situated in Shanghai, China

**DOI:** 10.1186/s12982-015-0033-8

**Published:** 2015-11-11

**Authors:** Joshua B. Mendelsohn, Liviana Calzavara, Lucia Light, Ann N. Burchell, Jinma Ren, Laiyi Kang

**Affiliations:** Dalla Lana School of Public Health, University of Toronto, Toronto, Canada; Ontario HIV Treatment Network, Toronto, Canada; Department of Family and Community Medicine, Li Ka Shing Knowledge Institute, St. Michael’s Hospital, Toronto, Canada; College of Medicine at Peoria, University of Illinois, Peoria, USA; Shanghai Centres for Disease Control and Prevention, Shanghai, China

**Keywords:** China, Shanghai, HIV, AIDS, Sexually transmitted infections, STI, STD, Prevention, Intervention, Workplace, Migration

## Abstract

**Background:**

China’s growing population of internal migrants has exceeded 236 million. Driven by rapid development and urbanization, this extreme population mobility creates opportunities for transmission of HIV and sexually-transmitted infections (STI). Large numbers of rural migrants flock to megacities such as Shanghai in search of employment. Although migrants constitute a key population at heightened risk of acquiring HIV or an STI, there is a lack of easily accessible sexual health services available for them. In response, we designed a short, inexpensive sexual health intervention that sought to improve HIV and STI knowledge, while reducing stigma, risky sexual behaviour, and sexual transmission of HIV and STI among migrant construction workers (MCW) situated in Shanghai, China.

**Results:**

We implemented a three-armed, community-randomized trial spread across three administrative districts of Shanghai. The low-intensity intervention included educational pamphlets. The medium-intensity intervention included pamphlets, posters, and videos. The high-intensity intervention added group and individual counselling sessions. Across 18 construction sites, 1871 MCW were allocated at baseline to receive one intervention condition. Among baseline participants, 1304 workers were retained at 3-months, and 1013 workers were retained at 6-months, representing a total of 579 person-years of follow-up. All workers, regardless of participation, had access to informational materials even if they did not participate in the evaluation. Overall outputs included: 2284 pamphlets distributed, 720 posters displayed, 672 h of video shown, 376 participants accessed group counselling, and 61 participants attended individual counselling sessions. A multivariable analysis of participation found that men (aOR = 2.2; 95 % CI 1.1, 4.1; p = 0.036), workers situated in Huangpu district (aOR = 5.0; 95 % CI 2.6, 9.5; p < 0.001), and those with a middle school education (aOR = 1.9; 95 % CI 1.2, 3.0; p = 0.01) were more likely to have participated in intervention activities.

**Conclusion:**

A brief educational intervention that prioritized ease of delivery to a highly mobile workforce was feasible and easily accessed by participants. Routine implementation of sexual health interventions in workplaces that employ migrant labour have the potential to make important contributions toward improving HIV and STI outcomes among migrant workers in China’s largest cities.

**Electronic supplementary material:**

The online version of this article (doi:10.1186/s12982-015-0033-8) contains supplementary material, which is available to authorized users.

## Background

In 2011, the Chinese Ministry of Health estimated that HIV prevalence had reached 780,000 cases, an 11 % increase over 2007 levels [1–3]. Syphilis incidence also increased from 17 to 32 cases per 100,000 persons over the same period [4, 5]. Although HIV and sexually transmitted infections (STI) remain concentrated among key populations, incidence is increasing within China’s general population [6–13]. China’s floating population (i.e., people who live outside of their “Hukou” area where their household is registered with the government) now exceeds 236 million persons, a number roughly equivalent to the total number of international migrants [14–17]. This mobile population has been referred to as a “bridging population” and a “tipping point” in relation to China’s HIV epidemic given their frequent separation from primary sexual partners, participation in transactional sex, and difficulty accessing HIV and STI prevention services [18–20]. These factors have been implicated in the expansion of opportunities for HIV and STI transmission within China [21–23]. Moreover, previous studies suggest that migrants may be more likely than non-migrants to use condoms inconsistently, report low levels of routine HIV testing, and to have casual sexual partners [6, 19, 20, 24–26]. Although the Hukou registration system was designed to facilitate the delivery of health and social services to persons registered and remaining within their home district, services for those who migrate to other districts are more limited [27–32]. HIV-related stigma creates additional barriers for migrants and other key populations in accessing sexual health services [8, 22, 33–37]. Therefore, there is a clear need for effective sexual health interventions targeted to migrant populations within China. Findings from prior intervention studies are, however, limited. In a recent systematic review of 16 studies conducted among floating populations in China between 2005 and 2012, sexual health interventions improved knowledge about HIV transmission and prevention and reduced stigma, but did not improve condom use behaviour [38]. Most of the included studies were uncontrolled trials, with some lacking good baseline measures. In construction, a common destination industry for migrant workers, contracts are typically 6–9 months in duration. When contracts end, workers move on to other projects that are often located in other cities. In China, there are thousands of similar worksites that reach millions of workers. Given the mobile nature of this population, short interventions and short study follow-up periods are required to minimize loss to follow-up. In response to these challenges, our objective was to design and evaluate a simple, brief and cost-effective sexual health educational intervention that sought to improve HIV and STI knowledge, while reducing HIV stigma, risky sexual behaviour, and new HIV and STI infections. Here, we document and report upon the design features, evaluation methods, and our implementation experiences in order to inform similar work in China and elsewhere.

This intervention was part of a 5-year collaboration between the Shanghai Centres for Disease Control and Prevention (Shanghai CDC) and the University of Toronto, which engaged governments, non-governmental organizations and members of vulnerable and at-risk groups including men who have sex with men, female sex workers, and migrant construction workers (MCW) in responding to the ongoing epidemic of HIV and STI in Shanghai. The “Prevention, care and support of migrant construction workers at risk for sexually transmitted infections (STI) and Human Immunodeficiency Virus (HIV) in Shanghai, China”  was one of the two intervention research components; the other was aimed at female sex workers. The aim of this report is to discuss the context, study design, implementation challenges, and recommendations that emerged from the sexual health intervention delivered to MCW.

## Methods

### Study setting

Shanghai, China’s second largest city of 24 million residents, is situated on the central eastern coast of China. The floating population who stayed for at least six months in Shanghai doubled to 9.6 million over the 5-year period ending in 2013 [39]. New HIV infections grew by 46 % between 2009 and 2011 (886–1294) with over two-thirds (68 %) occurring among rural-to-urban migrants [7, 40]. Free medical care was available to residents with proof of medical insurance, which was typically provided as a benefit of employment. Since migrant workers are employed intermittently, their employers do not usually pay for their insurance. The Chinese National HIV Program runs HIV testing sites that offer free testing, counselling and treatment (“Four Free and One Care”) [41]. In some provinces (including Shanghai) construction companies have been ordered to pay for temporary medical insurance to cover annual physical examinations, routine blood tests and chest X-rays.

### Study design

**Fig. 1:**
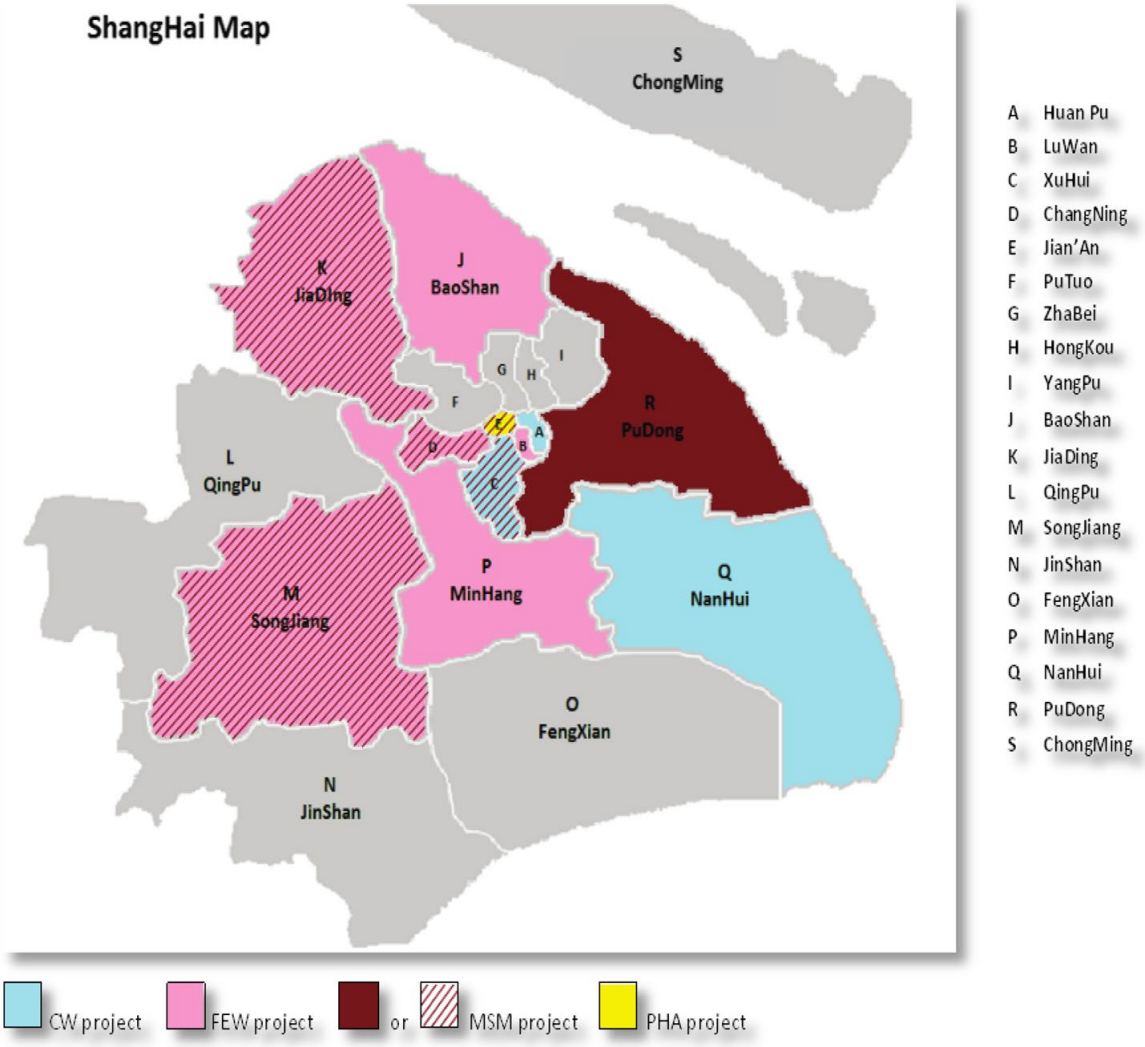
Map of Shanghai Districts involved in the Canada–China Program of HIV/STI research. Note 1: Districts with diagonal coloring participated in multiple projects under the same program of research. Note 2: When the Program was launched in 2007, there were 19 districts in Shanghai. Two districts merged in 2009 (Pudong and Nanhui) reducing the total number of districts to 17. *CW* construction workers, *FEW* female entertainment workers, *MSM* men who have sex with men, *PHA* people living with HIV and AIDS

We designed a three-armed community-randomized trial to evaluate a sexual health intervention implemented in 18 construction worksites spread across three districts of Shanghai (Fig. 1). Implementation of the trial was divided into three phases. In Phase 1, baseline data was collected from participating sites using an interviewer-administered survey and HIV/STI testing. In Phase 2, three community-based educational and behavioural interventions were implemented. A low-intensity intervention arm served as the control arm and consisted of distributing educational pamphlets. An intermediate-intensity intervention arm added educational posters, a poster exhibit and a series of educational videos. A high-intensity intervention arm added a counselling component consisting of group and individual counselling sessions. In Phase 3, we conducted follow-up interviews at 3- and 6-months to assess the impact on sexual risk behaviours, HIV and STI risk, knowledge, and stigma. We repeated STI testing at 6-months follow-up.

### Eligibility, randomization and power calculation

In selecting districts, construction sites and individual participants, we employed purposive and random sampling methods tailored to the study context. Districts were purposively selected, while venues and individual MCW were randomly sampled. All 19 districts in Shanghai were classified as central urban (n = 9), inner suburban (n = 4) or outer suburban (n = 6) areas according to their location within the city. The districts of Huangpu, Xuhui, and Nanhui were purposively selected by the Research Team to represent each category. Each district health authority provided a list of active construction sites. Construction site managers were canvassed with regards to their willingness to participate. To be eligible, construction site managers had to declare a reasonable expectation that most of their workforce would be retained after six months and at least 50 workers would be employed at the site by the start of the intervention (i.e., so that the study could attain sufficient power). All active construction sites within each district were assessed against these eligibility criteria. The Research Team then randomly selected six construction sites within each of the three pre-selected districts (n = 18) and randomly allocated two construction sites per district to each intervention arm (n = 6 per arm). Randomization of construction sites was accomplished by assigning a unique identifier to each site from a set of random numbers which were then sorted in ascending order. Sites were approached until the desired quota (n = 6) was reached within each district. If a selected site refused, it was replaced by the following site as indicated on the ordered list. A list of MCW was provided by each participating construction site manager. From this list, a random sample of MCW were approached by the Field Team. At smaller sites, all MCW were approached until the required sample size was achieved. All workers employed within each construction site at the time of the intervention were eligible to participate in the study if they were ≥18 years old and gave informed consent.

The primary outcome used to evaluate impact was the proportional change in those reporting unprotected sex with a non-spousal partner. The expected effect size was a proportional decrease of 50 %, from 15 to 7.5 % in absolute prevalence. This effect was consistent with a review published by Crepaz et al. which found that the effect of similar preventive interventions resulted in a halving of the odds of risky sexual behaviour (OR = 0.57; 95 % CI 0.4, 0.82) [42]. Without previous data to estimate the coefficient of variation between clusters, k, Hayes & Bennett advise that most field trials can employ k ≤0.25, with plausible values of k used to examine potential sample size estimates [43]. Therefore, assuming a value of k = 0.25, 80 % power and p = 0.05, we calculated that a two-arm trial would require a minimum of four clusters (i.e., construction sites) per arm, with a minimum of n = 50 participants per cluster. Given that we planned to conduct a three-arm trial, and taking into account possible attrition of sites and participants, we conservatively increased the number of clusters to six per arm and the minimum number of participants to 100 per cluster. Secondary outcomes included: (1) HIV/STI knowledge; (2) HIV/STI stigma, and (3) condom knowledge and use; and (4) prevalence of HIV and STI infections after six months of follow-up.

### Intervention design

The study, conducted between June 2008 and January 2009, included interventions which lasted between 1 and 2 weeks at each construction site. Interventions were delivered during working hours and were initiated upon completion of baseline interviews at each construction site. All employees had access to printed educational materials and videos regardless of their participation in the evaluation component (Table 1).

**Table 1:** Summary of intervention activities

	Low-intensity intervention	Intermediate-intensity intervention	High-intensity intervention
Educational component	Pamphlets	Pamphlet; posters; videos	Pamphlet; posters; videos
Behavioural component^a^	–	–	Group counselling and discussion; one-on-one counselling
Allocation	Six randomly selected construction sites	Six randomly selected construction sites	Six randomly selected construction sites
Access	All workers at the site	All workers at the site	All workers at participating sites, regardless of participation in the study, had access to the educational component but only those who agreed to participate in the study were offered the counselling intervention. All participants who agreed to participate in the high-intensity intervention were allocated to group discussion. Additional one-on-one sessions were given upon request

^a^All workers who agreed to undergo HIV/STI testing and who tested positive were provided with one-on-one counselling regardless of the intervention arm they were randomly selected to receive

The intervention and evaluation protocols were developed in consultation with the Shanghai CDC, their administrative health representatives at the district-level in Shanghai, and researchers at the Dalla Lana School of Public Health, University of Toronto. A Study Advisory Committee (SAC) consisting of migrant workers, construction site managers, officials from the Shanghai CDC, the Office of Migrant Management, the Bureau of Health, health care providers, and migrant health researchers, collectively provided guidance and oversight. The Research Team consisted of health service providers and officials from health districts. Recruitment, baseline interviews, and HIV and STI tests were administered by a Field Team comprised of Shanghai CDC health workers. Participants were offered free treatment if clinically indicated after HIV and STI testing.

Intervention materials were selected from pre-existing stocks after a thorough review of national, municipal, and district-level HIV and STI educational materials and recommendations from prior sexual health interventions conducted in China and elsewhere [44–46]. Materials were then reviewed by the Research Team, revised, and approved by the SAC. A manual of standard operating procedures was developed to facilitate training and to ensure that intervention delivery was standardized across worksites. Construction site managers and district-level CDC health workers also received training in HIV and STI prevention. Ten healthcare providers from each of the three participating districts were recruited and trained for at least 30 h to implement and evaluate the intervention. The health educators who were selected to facilitate the group and individual counselling sessions for the high-intensity intervention arm received additional training. Facilitators were provided with a list of questions and themes to discuss with participants and a copy of relevant reference material to help ensure that answers to factual questions were correct and consistent. All participants received a card with instructions on where to direct questions or concerns, or to inform the Research Team if they would be leaving the construction site before their next interview date.

### Low-intensity intervention arm

The low level intervention consisted of educational pamphlets, entitled “HIV/AIDS: How Much Do You Know?” and three STI pamphlets with information on chlamydia, gonorrhoea, and syphilis (Additional file 1: Figure S1). The pamphlets used cartoon images to convey information, an example of which included a scene where a male and a female cartoon condom are discussing the importance of condom use. Pamphlet text provided basic information on HIV/AIDS and STI including symptoms, testing, disease progression, transmission, treatment, and prevention, and additional resources. Pamphlets were also made available in public spaces (i.e., cafeteria, meeting rooms, and worksite medical clinics) where workers who declined to participate or were not selected to participate in the evaluation could have voluntary access to the pamphlet.

### Intermediate-intensity intervention arm

The intermediate level intervention consisted of posters, movable poster displays, and educational videos, in addition to the pamphlets used in the low-intensity intervention. Sixty large posters, entitled “Wear a Helmet for Safety; Wear a Condom for Good Health” (Additional file 2: Figure S2) were displayed within the constructions sites allocated to the intermediate or high-intensity arms (N = 720 large posters). One movable poster display consisted of six panels (e.g., four panels dedicated to HIV, two panels for STI) and were rotated around communal areas. Posters and movable poster displays were exhibited in the dining hall, dormitories, outside meeting rooms, and outside the medical clinic. These materials conveyed educational messages regarding modes of HIV and STI transmission, misunderstandings about modes of HIV/AIDS transmission, signs and symptoms of HIV/AIDS, the benefits of HIV/STI testing and treatment, HIV prevention, condom use, and stigma. Additionally, a series of nine short videos of 3–5 min in duration were shown in a loop that lasted 40 min per cycle, for a total of 8 h per intervention-day. The videos were shown in the worksite dining hall and activity rooms during meal times. The video was introduced by a trained member of the Research Team who was available to answer questions during and after viewings. The videos used personal stories and expert information to educate MCW about HIV/AIDS testing, living with AIDS, the harms of HIV/AIDS stigma, sexual transmission, injection-drug use, mother-to-child transmission, and condoms. By way of example, one video portrayed a young heterosexual man who met a woman on the internet after breaking up with his girlfriend; a second video depicted a woman with a supportive family and an uninfected husband who spoke about the importance of condoms in their sexual relationship.

### High-intensity intervention arm

The high-intensity arm added group and individual counselling sessions to the intervention activities included in the other arms. Each study participant took part in one group counselling session and was offered an optional individual counselling session. Each group session was limited to five workers, lasted approximately 60 min, and was held in private meeting rooms. To increase comfort levels, participants were grouped by home village and gender. At the start of the session, a verbal promise was obtained from each participant indicating that they would not reveal any information disclosed by other participants during the session. At the end of each session, health care workers were available to address private questions and concerns. The group sessions took place during workers’ time off, typically in the evenings, and were structured to cover a set of standardized topics while allowing for flexibility to address issues raised by participants. The individual sessions did not follow a structured format and were used to facilitate in-depth discussions of topics covered in the group sessions or to ask private questions. In the worksites allocated to the high-intensity arm, the posters, pamphlets and videos were also available to all workers, but group counselling sessions were limited to study participants.

### Ethical considerations

Ethical approvals were obtained from the University of Toronto (Approval #21866) and the Independent Ethics Board of the Shanghai Municipal Centre for Disease Control and Prevention Institutional Review Board (Approval #IORG00000630). Participation in the study was voluntary and participants were free to withdraw at any time without penalty. Participants provided individual consent for each type of data collected (e.g., survey, biological specimens). Biological specimens were collected by the Field Team. The Field Team was also responsible for providing pre-test counselling. Participation incentives included access to free and confidential HIV and STI testing and treatment, free medical services including a medical examination, testing for Hepatitis B, malaria, schistosomiasis, and tuberculosis, a package of personal hygiene items worth USD $3.00 in local currency (equivalent to an average hourly wage), and refreshments during intervention sessions. Participating construction sites were provided with an honorarium of $500 USD.

## Results

### Health outcome assessment

#### Questionnaire

In the design phase (Phase 1), a questionnaire was prepared in English and translated separately by two teams of translators into Mandarin. These versions were compared, discussed and revised for consistency with the original English version. The questionnaire was pre-tested on a sample of construction workers from three worksites that were not included in the main study. The survey took between 30 and 60 min and responses were recorded on paper questionnaires. The primary outcome, unprotected sex with non-spousal partners, was derived from an indicator for consistent condom use with each partner type. Self-reported condom use was measured on a five-point Likert scale (i.e., “never”, “rarely”, “sometimes”, “most of the time”, or “all of the time”). HIV knowledge was measured as the sum of ten questions with binary response options regarding actual and misunderstood modes of transmission including shared needles, unprotected sex, multiple sex partners, mother-to-child transmission, mosquitoes, coughing, kissing, shaking hands, sharing food, and sharing towels or toilet facilities with an HIV-positive person. HIV stigma was measured as level of agreement, on 4-point Likert scales, to three statements about people living with HIV and AIDS: (1) “they do not deserve sympathy”; (2) “they are scary”; and (3) “they are disgusting”. An additive formula of each 4-point Likert scale was used to derive a summary stigma score of 0–9. This measure was adapted from a study previously conducted among rural–urban migrants in China [47]. Beliefs about condom effectiveness and knowledge of correct use were measured as the sum of six questions evaluating agreement, on a 5-point Likert scale, to statements regarding condom use, condom attitudes (e.g., “using condoms is a good contraceptive measure”) and condom availability (e.g., “condoms are easy to obtain”).

#### HIV and STI testing

Participants provided whole blood specimens (5 mL) at baseline to screen for HIV, syphilis and herpes simplex type 2 (HSV-2). Blood was tested with enzyme immunoassays (ELISA) for HIV (Livzon, Guangdong, China) with positive tests confirmed by Western Blots (MP Biomedicals, Santa Ana, USA). For syphilis, blood was tested with rapid plasma reagin (RPR) (Keihua, Shanghai, China) and Treponemal palladium hemagglutination assay (TPHA) (Fujibiro, Tokyo, Japan and marketed by Livzon, Guangdong, China). Blood was tested for HSV Type 2 IgG antibodies using ELISA (Gaoda, Beijing, China). Urine specimens (15 mL) were collected at baseline and 6-months to screen for chlamydia and gonorrhoea. Urine samples were tested via nucleic acid amplification tests for Neisseria gonorrhoea (NG), Chlamydia trachomatis (CT) and Trichomonas vaginalis (TV) (Roche Diagnostic Systems, Branchburg, USA). In Chinese culture, blood is seen as a life-force. In the opinion of the local district CDC, the burden on the population of giving blood would be off-set by the benefit of being screened and treated for an infection which otherwise would go undetected and untreated at baseline. Given the low infection rate detected at baseline, however, a second blood specimen was thought to be overly burdensome and only chlamydia and gonorrhoea were tested at 6-months of follow-up (Table 2).

**Table 2:** HIV and STI testing conducted during baseline and follow-up

	Testing procedure	Required for study	Type of specimen	Collected/conducted at baseline	Collected/conducted at six months
Chlamydia	LCx	Yes	Urine	Yes	Yes
Gonorrhoea	LCx	Yes	Urine	Yes	Yes
Herpes simplex II	EIA	Yes	Blood	Yes	No
Syphilis	RPR, TPPA (if reactive)	Yes	Blood	Yes	No
HIV	EIA and Western blot (if repeatedly reactive)	Yes	Blood	Yes	No
Optional					
Medical exam	Routine examination	No	–	Yes	No
Tuberculosis	Tuberculin skin test	No	X-ray^a^	Yes	No

*LCx* nucleic acid amplification assay, *EIA* enzyme immunoassay, *RPR* rapid plasma reagin, *TPPA* Treponema pallidum particle agglutination assay

^a^If deemed necessary by healthcare provider

### Process evaluation

A range of activities related to infrastructure, human resources and quality control were undertaken to ensure the intervention was implemented and evaluated consistently. The Shanghai CDC built a designated project office for sexual health in order to manage and implement this study. The project office selected three districts and appointed the Heads of the Department of HIV/AIDS Control as the Study Coordinators for each district. The Study Coordinators recruited the Field Team, who were responsible for implementing the intervention and collecting data during the evaluation phase. All partner institutions (University of Toronto, the Shanghai CDC and local district CDC branches) shared responsibility for intervention quality control. The SAC was responsible for monitoring the intervention, providing feedback on fidelity to protocol, and making recommendations to improve delivery. Study Coordinators were responsible for overseeing intervention roll-out, data collection, and ensuring consistency of delivery across construction sites.

Quality control was based on detailed notes produced by the Field Team regarding daily intervention activities. These notes were relayed to Study Coordinators who reviewed procedures and provided feedback to the Research and Field Teams. Study Coordinators also conducted four unannounced inspections over the duration of the intervention to check on fidelity to protocol. The construction site managers' role consisted of facilitating access to the construction site by the Field Team and helping to set up materials. Managers received some basic training in HIV/STI prevention alongside the Field Team. They had no direct role in intervention delivery, i.e., they were not delivering counselling sessions or answering questions after video sessions. In total, 4259 interviews were conducted, 2284 pamphlets were distributed, 2183 urine specimens were collected, 1574 blood samples were collected, 720 posters and 12 movable poster exhibitions were displayed, 672 h of video were shown, 509 training sessions were attended, 376 participants were counselled in groups (representing 25–50 % of all workers at participating worksites), and 61 participants were counselled individually. Additionally, issues related to migration and attrition were documented and informed Research Team debriefing sessions.

**Fig. 2:**
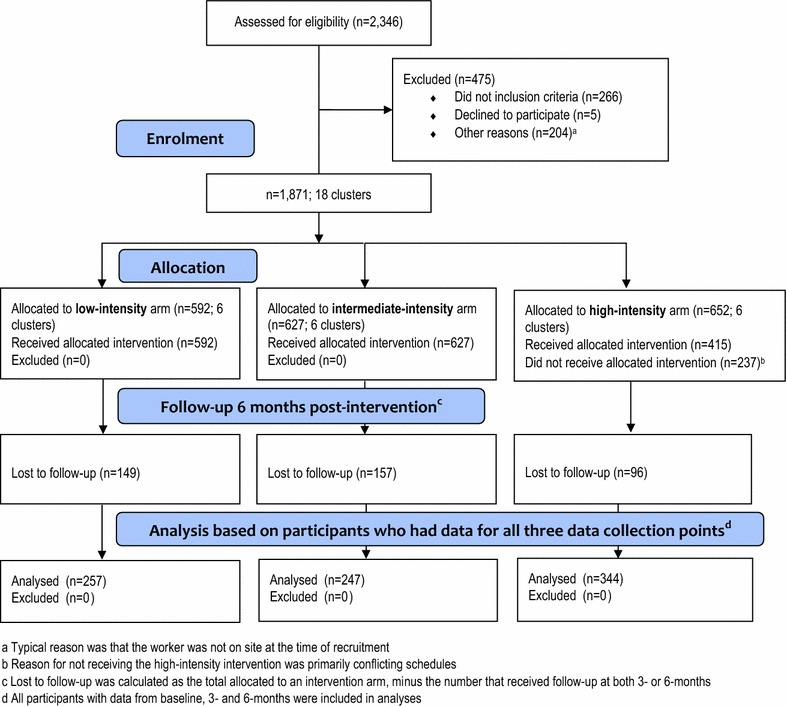
Study flow diagram

The results of recruitment and participation are outlined in Fig. 2. Of the 26 pre-identified construction sites within the three participating districts, 18 sites were randomly selected. All sites were approached and 100 % agreed to participate. At the time of recruitment, of the 3638 workers on the employee lists at the 18 sites, 2346 were randomly selected for participation. Of these, 1871 were present at the time of recruitment and agreed to participate. Therefore, in addition to the 1871 evaluation participants, 1767 non-participating MCW who were employed across the 18 construction sites also had access to basic intervention materials. Interviews at 3- and 6-months accounted for 579 person-years of follow-up. Attrition between baseline and 6-months follow-up was 46 %. Participating construction sites employed a range of 57–179 workers over the duration of the intervention period. The participation rate among construction workers who were approached about the study was 99 %. We compared trial arms on sociodemographic characteristics (Table 3), finding differences in age, years married or cohabiting, gender, partner visits in the past year, lodging, province of birth, years migrating, number of cities migrated to, STI symptoms in the past year (all p < 0.001), marital status (p = 0.005), and years spent in Shanghai (p = 0.002). These factors will be adjusted for in main analyses.

**Table 3:** Socio-demographic characteristics of participants across intervention arms at baseline (n = 1871)

Characteristic	Total, n (%)^a^	Low-intensity/control, n (%)	Intermediate-intensity, n (%)	High-intensity, n (%)	*p* value*
Age (mean years, SD; n = 1850)	38.7 (10.4)	38.5 (11.1)	37.2 (10.6)	40.5 (9.3)	<0.001**
Marriage/cohabitation time (mean years, SD; n = 1538)	16.8 (9.0)	16.7 (9.6)	15.6 (9.1)	18.0 (8.3)	<0.001**
Gender (n)	1871				
Male	1757 (93.9)	531 (30.2)	591 (33.6)	635 (36.1)	<0.001
Female	114 (6.1)	61 (53.5)	36 (31.6)	17 (14.9)	
Marital status (n)	1870				
Married/living together	1556 (83.2)	484 (31.1)	505 (32.5)	567 (36.4)	0.005
Single/separated	314 (16.8)	107 (34.1)	122 (38.9)	85 (27.1)	
Partner visits, past year (n)	1073				
1–3	243 (22.6)	80 (32.9)	62 (25.5)	101 (41.6)	0.001
4+	79 (7.4)	26 (32.9)	29 (36.7)	24 (30.4)	
Living with Partner	424 (39.5)	168 (39.6)	141 (33.3)	115 (27.1)	
Single or did not visit partner	327 (30.5)	109 (33.3)	125 (38.2)	93 (28.4)	
Lodging (n)	1869				
Dormitory	1568 (83.9)	532 (33.9)	450 (28.7)	586 (37.4)	<0.001
Rent apartment	242 (13.0)	36 (14.9)	150 (62.0)	56 (23.1)	
Own house	35 (1.9)	14 (40.0)	18 (51.4)	3 (8.6)	
Other	23 (1.2)	8 (34.8)	8 (34.8)	7 (30.4)	
Education (n)	1870				
Elementary or less	460 (24.6)	163 (35.4)	151 (32.8)	146 (31.7)	0.163
Middle school	1051 (56.2)	330 (31.4)	352 (33.5)	369 (35.1)	
High School or more	359 (19.2)	99 (27.6)	123 (34.3)	137 (38.2)	
Province of birth (n)	1864				
Jiangsu	612 (32.8)	111 (18.1)	179 (29.3)	322 (52.6)	<0.001
Anhui	338 (18.1)	109 (32.3)	115 (34.0)	114 (33.7)	
Sichuan	336 (18.0)	91 (27.1)	133 (39.6)	112 (33.3)	
Other	578 (31.0)	279 (48.3)	199 (34.4)	100 (17.3)	
Years migrating (n)	1818				
< 5	362 (19.9)	121 (33.4)	150 (41.4)	91 (25.1)	<0.001
5–9	353 (19.4)	100 (28.3)	144 (40.8)	109 (30.9)	
10–14	389 (21.4)	130 (33.4)	120 (30.9)	139 (35.7)	
15+	714 (39.3)	221 (31.0)	186 (26.1)	307 (43.0)	
Number of cities migrated to (n)	1822				
0–1	387 (21.2)	156 (40.3)	134 (34.6)	97 (25.1)	<0.001
2–4	1128 (61.9)	323 (28.6)	360 (31.9)	445 (39.5)	
5+	307 (16.8)	96 (31.3)	105 (34.2)	106 (34.5)	
Years in Shanghai (n)	1814				
< 1	361 (19.9)	90 (24.9)	136 (37.7)	135 (37.4)	0.002
1–4	433 (23.9)	144 (33.3)	160 (37.0)	129 (29.8)	
5–9	363 (20.0)	114 (31.4)	114 (31.4)	135 (37.2)	
10+	657 (36.2)	222 (33.8)	188 (28.6)	247 (37.6)	
STI symptoms past year, self-report (n)	1860				
Yes	155 (8.3)	65 (41.9)	56 (36.1)	34 (21.9)	<0.001
No	1705 (91.7)	524 (30.7)	568 (33.3)	613 (36.0)	
Ever tested for HIV (n)	1833				
Yes	21 (1.2)	6 (28.6)	7 (33.3)	8 (38.1)	0.931
No	1812 (98.9)	576 (31.8)	610 (33.7)	626 (34.6)	
Lifetime sexual partners ≥1 (n)	1706				
1	1315 (77.1)	421 (32.0)	412 (31.3)	482 (36.7)	0.171
2	206 (12.1)	62 (30.1)	80 (38.8)	64 (31.1)	
3+	185 (10.8)	57 (30.8)	68 (36.8)	60 (32.4)	

* Chi squared test, ** Kruskal–Walllis test

^a^Except for age and marriage/cohabitation time

At the 3-month follow-up visit, 79 % of participants across all intervention arms reported engaging with at least 75 % of the intervention materials. We created an intervention participation outcome that was dichotomized to represent participation in ≥50 % or <50 % of intervention activities. For example, in the low-intensity intervention there were two pamphlets; if a participant reported reading only one, we assigned a score of 0.5. If they reported reading both, a score of 1 was assigned. Similarly, for the intermediate- and high-intensity intervention arms, the denominator consisted of four and six materials, respectively. After adjusting for age, district, education, and intervention level, men were twice as likely as women to participate in intervention activities (aOR = 2.2; 95 % CI 1.1, 4.1; p = 0.036). There was no evidence for any differences in participation between younger (<30 years old) and older MCW (aOR = 1.1; 95 % CI 0.6, 1.9; p = 0.766). MCW who worked in Huangpu district were five times more likely to engage than workers situated in Xuhui district (aOR = 5.0; 95 % CI 2.6, 9.5; p < 0.001). More educated workers had higher odds of participating than less educated workers (aOR = 1.9; 95 % CI 1.2, 3.0; p = 0.01). The effect of education on participation in intervention activities was weakened slightly when comparing workers with a high school education or greater, to an elementary level or less (p = 0.058). Intervention level was not associated with participation in intervention activities (Table 4).

**Table 4:** Adjusted odds ratios for ≥50 % or <50 % participation in intervention activities

Variable (n)	Unadjusted			Adjusted		
	Odds ratio	95 % confidence interval	p value	Odds ratio	95 % confidence interval	p value
Gender						
Female (80)	1.0			1.0		
Male (1209)	2.6	1.4, 4.8	0.004	2.1	1.1, 4.1	0.036
Age (years)						
<30 (238)	1.0			1.0		
≥30 (1046)	0.9	0.5, 1.6	0.79	1.1	0.6, 1.9	0.766
District						
Xuhui (393)	1.0			1.0		
Huangpu (430)	5.2	2.7, 9.9	<0.001	5.0	2.6, 9.5	<0.001
Nanhui (466)	1.8	1.2, 2.9	0.009	2.1	1.3, 3.4	0.002
Education level						
Elementary or less (303)	1.0			1.0		
Middle school (724)	2.1	1.3, 3.2	0.002	1.9	1.2, 3.0	0.01
High school or more (261)	2.3	1.2, 4.3	0.01	1.9	1.0, 3.7	0.058
Intervention arm						
Low (384)	1.0			1.0		
Intermediate (427)	1.6	1.0, 2.6	0.067	1.5	0.9, 2.5	0.142
High (480)	1.8	1.1, 3.0	0.02	1.6	0.9, 2.6	0.101

Reflecting upon lessons learned throughout design and implementation of this intervention, we suggest four key points to consider when developing interventions for migrant, mobile, and short-term workers. First, studies with short follow-up periods are highly susceptible to attrition. Therefore, cultural festivals and vacation periods that could result in home visits or travel for participants during the intervention or evaluation period should be identified well in advance. This oversight could preclude recruitment of sufficient numbers of participants while potentially introducing bias stemming from differences between workers who are able to make return trips home and those who are not. Data collection in China was completed between June and January before Chinese New Year when many migrant workers make the journey back to their hometowns during the holiday period. Second, given that worksites are temporary and exist until a project completion date, the development of the cluster sampling frame may be best undertaken as close to the planned intervention start date as possible to ensure that all eligible worksites are properly represented within the sampling frame. Third, due to departure immediately after work hours and increased travel burden compared with workers who live on-site, workers who live offsite may have less engagement with the intervention activities given they forego the opportunity to browse materials during leisure time after working hours. In our case, 16 % lived off-site. Attempts to facilitate equivalent access for this group should be undertaken. Fourth, the duration of the typical contract length needs to be carefully assessed to account for attrition in power calculations. Short contracts may increase attrition over the designated follow-up period. These points are summarized in Box 1.

**Table:** 

Box 1: Lessons learned in implementing an education intervention among migrant construction workers
1. Any local or national festivals that could result in home visits or travel for participants during the intervention or evaluation should be identified well in advance. Such events could increase attrition and bias results. In our case, data collection in China was completed between June and January before home visits begin for Chinese New Year.
2. Given that worksites are temporary and exist until a project completion date, the development of the sampling frame needs to be undertaken as close to the planned start date of the intervention as possible in order to ensure that eligible worksites are properly represented within the sampling frame.
3. Consider any potential biases introduced by workers who live offsite and therefore engage less with intervention activities, or may be less well represented in the sample. For example, offsite workers may face an increased travel burden which may limit their engagement in relation to other groups.
4. Consider the duration of the typical worker contract and how this might affect loss to follow-up. Short contracts may increase attrition over the designated follow-up period, which may introduce biases when comparing groups.

## Discussion

The combined effects of urbanization, economic restructuring and welfare reforms in China have fueled a massive relocation from rural to urban areas. Despite the 2004 New Rural Cooperative Medical Scheme and the 2009 National Health Reform which were intended to reduce health outcome disparities while improving accessibility of services [33], migrant workers in China continue to face barriers when accessing sexual and reproductive health services [48]. The Canada-China program of intervention research with MCW was undertaken in response to the vulnerability of migrant workers to HIV and STI infections [1–5]. He and colleagues found that 87 % of married male construction workers situated in Shanghai did not live with their wives, compared with 39 % of factory workers and 11 % of market vendors; such lengthy separations are associated with increases in STI transmission [23, 49]. HIV and STI-related stigma within communities of migrant workers exacerbate barriers to self-protective behaviours including HIV and STI testing, treatment, and spousal notification of infection [50, 51].

Our intervention, designed for short-term and frequent delivery to migrant workers, benefitted from pre-existing materials and human resource capacity. This lowered costs while simplifying implementation so that the intervention could be delivered and evaluated within the brief windows of time before migrant workers moved onto to new jobs. Our intervention and evaluation was designed for routine implementation over 3–6 months in order to accommodate this rapid turnover. This intervention also drew upon extant productive collaborations between the Shanghai CDC, district-level health workers and construction site managers, in order to legitimize the intervention among stakeholders and to quickly and effectively integrate the intervention into daily work schedules. While direct engagement of site managers and local health district workers in implementation required a greater up-front investment in training, our hope was to facilitate uptake and sustainability beyond the research phase.

After comparing participants on sociodemographic characteristics, we found significant differences in age, years married or cohabiting, gender, partner visits in the past year, lodging, province of birth, years migrating, number of cities migrated to, STI symptoms in the past year, marital status, and years spent in Shanghai. These differences were likely due to random chance given the small number of clusters within each arm and highlight the importance of adjusting for these factors when comparing intervention conditions. In analyses that evaluated participation in intervention activities, there was no association between intervention arm and participation. Men, those with a middle school education, and workers based in Huangpu and Nanhui districts were more likely to engage with intervention activities. Female MCW were less likely to participate than their male counterparts. One reason may be that women were a small minority at participating construction sites, and may have chosen not to spend time in communal areas where most intervention activities took place. A more targeted approach to sexual health education for female MCW is necessary. It is unclear why workers in Xuhui were less likely to participate than workers from other districts. Additional work that aims to identify social and structural factors within districts that might complicate intervention delivery is needed. We found a strong association between middle school education and participation, but a weaker association when considering high school education. Taken together, the finding that educated workers were more likely to participate suggests that future interventions should ensure that materials are easily understood and accessed by less educated workers. We evaluated participation in intervention activities but did not complete a detailed assessment of how literacy may have affected comprehension of intervention materials. A number of steps were taken to minimize the impact of illiteracy. We administered questionnaires in face-to-face interviews to avoid comprehension difficulties that might have introduced bias if it had been self-completed. We aimed to bolster comprehension by including images (i.e., pamphlets and posters), multimedia (i.e., videos), and engagement (i.e., counselling sessions).

Limitations of the study design included the compressed timeframe in which to evaluate impact and high levels of attrition. Attrition between baseline and 6-months follow-up was 46 %. Methods to increase numbers of migrant workers present at follow-up need to be explored in future studies. Some MCW (~16 %) lived off-site in rented apartments, houses, or other external accommodations. For these workers, departure from the construction site in the evenings may have reduced the amount of time they had to engage with intervention activities before or after shifts. Place of residence should therefore be adjusted for in main analyses.

A strength of the study was our Research Team, which was comprised of healthcare providers recruited from district branches of the Shanghai CDC. Their prior training and familiarity with district health services facilitated high quality and consistent implementation and data collection across construction sites while reducing the overall cost of implementation (i.e., their time was paid as an in-kind contribution by the district branches of the Shanghai CDC). However, hierarchical social structures and perceptions of socially acceptable behaviour in China may have introduced social desirability bias in relation to sensitive questions regarding sexual behaviour. A second strength was the engagement of construction site managers and local health workers, which helped to facilitate the adoption and sustainability of intervention programs. Finally, the short timeframe in which the intervention was implemented and evaluated constituted an important strength of this work in relation to the inherent challenges of studying migrant workers.

In summary, a sexual health intervention designed to increase HIV and STI knowledge while reducing sexual risk, stigma and new infections, was designed and implemented among highly mobile migrant construction workers in Shanghai, China. In responding to evidence gaps in relation to sexual health interventions among this population, we reported upon design features, evaluation methods, and our implementation experiences. In China, there are thousands of similar worksites that reach millions of workers. Our intervention could be adapted in other settings situated in China and elsewhere that are characterized by highly mobile workforces in need of HIV and STI prevention interventions.

## Additional files

10.1186/s12982-015-0033-8 Sample educational pamphlet used as core curriculum in an intervention conducted among migrant construction workers in Shanghai, China.
10.1186/s12982-015-0033-8 Sample “large” (42 × 60 inches) poster used to promote an intervention among migrant construction workers in Shanghai, China.
