# Supplementary material for: Design and implementation of a sexual health intervention for migrant construction workers situated in Shanghai, China
Source: Emerg Themes Epidemiol. 2015 Nov 11;12:16. doi: 10.1186/s12982-015-0033-8 (PMC4642640; doi:10.1186/s12982-015-0033-8)
Supplement: Supplementary file 1 — 10.1186/s12982-015-0033-8 Sample educational pamphlet used as core curriculum in an intervention conducted among migrant construction workers in Shanghai, China. [file 12982_2015_33_MOESM1_ESM.docx]

Figure 1: Sample educational pamphlet used as core curriculum in an intervention conducted among migrant construction workers in Shanghai, China

| **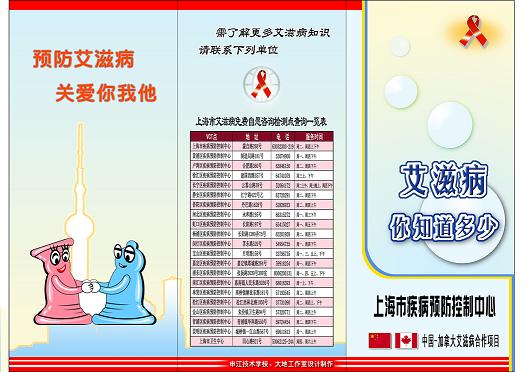** | **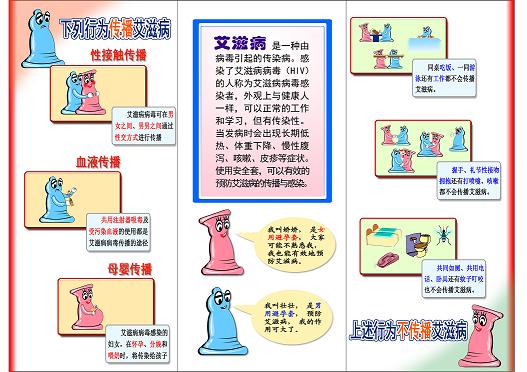** |
| --- | --- |
| **Panel A (Front of pamphlet)**  **Panel 1**: Condom couple  Title, Line 1-2: Two condoms holding hands, Pearl of Asia Tower in the background “AIDS Prevention; Caring for yourself and others”  **Panel 2:** AIDS ribbon above pink and white table  Title, Line 1-2: “If you would like to know more about AIDS, please contact the below places”  Table heading, Line 3: “Shanghai Voluntary AIDS Testing and Counseling Information”  Table content: Names, addresses, telephone numbers, and hours of operation of all the local district Centers of Disease Control and the Shanghai Centre of Disease Controls main number  **Panel 3:** AIDS ribbon in the corner, bubble background, Canadian and Chinese flags in bottom left corner  Title, Line 1-2: “How much do you know about AIDS?”; Line 3: “Shanghai Centre for Disease Prevention and Control”; Line 4: “China-Canada Collaborative Project” | **Panel B (Back of pamphlet)**  **Panel 3**: Sad blue condom in top left corner, picture of two condoms hugging, picture of male condom using a needle, picture of female condom pregnant  Title, Line 1: “The following are behaviors that transmit AIDS”  Text for diagram 1 (hugging couple), Title, Line 2: “Transmission through sexual contact”; Bubble 1, Line 3-5: “AIDS can be transmitted through heterosexual intercourse and male to male sexual intercourse”  Text for diagram 2 (injecting condom), Title, Line 6: “Transmission through blood”; Bubble 2, Line 7-9: “Sharing needles and syringes while doing drugs and using contaminated blood are ways to contract AIDS”  Text for diagram 3 (pregnant condom), Title, Line 10: “Transmission between mother and child”; Bubble 3, Line 11-13: “A pregnant HIV positive woman can transmit the virus to her child during pregnancy, child birth, and/or breastfeeding”  **Panel 4:** Blue and pink textbox, pink condom speaking with blue condom below also speaking  Title, Line 1: AIDS;  Textbox, Line 1-12: “AIDS is an infectious disease caused by a virus. Those who are infected with HIV are called HIV carriers and they look the same as a healthy person. They can go to work and go to school, but they are infectious. When they begin to show symptoms they will suffer from a chronic low grade temperature, weight loss, diarrhea, a cough, skin lesions, etc. Use a condom to effectively prevent AIDS transmission and infection.”  Pink Condom Bubble, Line 13-17: “My name is ZhaoZhao and I am a female condom. You may not be familiar with me, but I am also effective in preventing AIDS.”  Blue Condom Bubble, Line 18-21: “My name is ZhuangZhuang. I am a male condom. I am very effective in preventing AIDS.”  **Panel 5:** Three panels full of various activities that do not transmit HIV/AIDS; pink female condom in bottom right corner.  Title, Line 13, bottom of the page: “The above behavior will not transmit AIDS”  Diagram 1 Text, Line 1-3: “Eating together at the same table, swimming together, and working together will not transmit AIDS”  Diagram 2 Text, Line 4-6: “Shaking hands, greeting someone with a kiss, hugging, sneezing and coughing will not transmit AIDS”  Diagram 3 Text, Line 7-9: “Using the same washroom, telephone, and bed, as well as, mosquito bites will not transmit AIDS” |
