# Supplementary material for: Design and implementation of a sexual health intervention for migrant construction workers situated in Shanghai, China
Source: Emerg Themes Epidemiol. 2015 Nov 11;12:16. doi: 10.1186/s12982-015-0033-8 (PMC4642640; doi:10.1186/s12982-015-0033-8)
Supplement: Supplementary file 2 — 10.1186/s12982-015-0033-8 Sample “large” (42 × 60 inches) poster used to promote an intervention among migrant construction workers in Shanghai, China. [file 12982_2015_33_MOESM2_ESM.docx]

Figure 2: Sample “large” (42 x 60 inches) poster used to promote an intervention among migrant construction workers in Shanghai, China

| 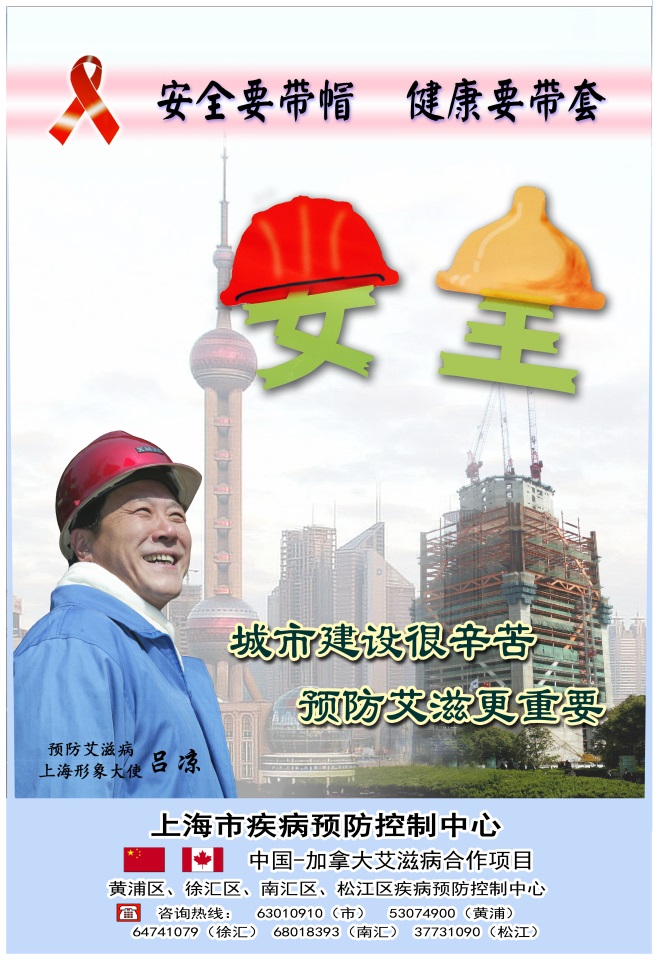 | 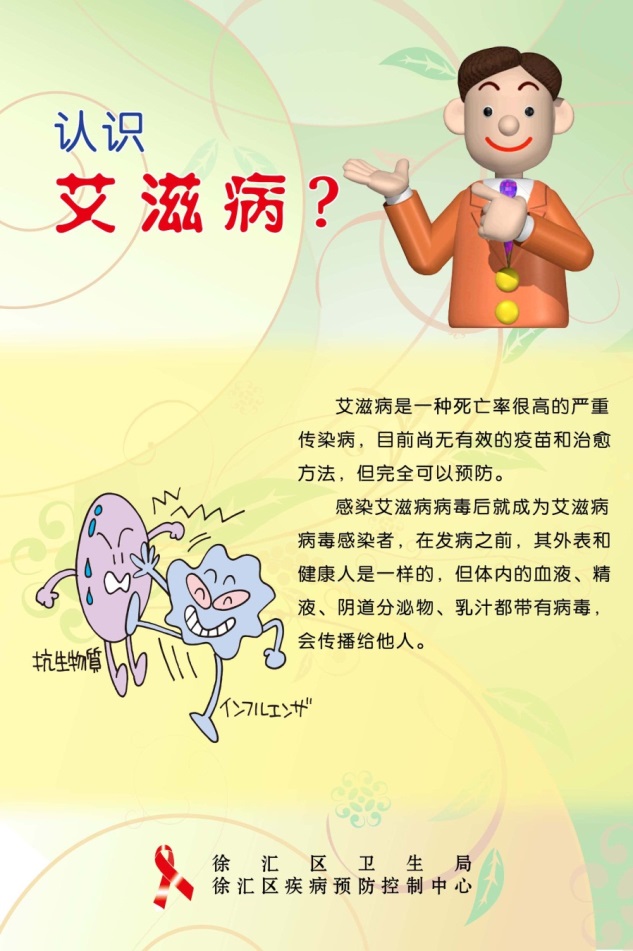 |
| --- | --- |
| **Main Project Poster:** Smiling Man in Hard Hat  **Top Poster Slogan**: For safety wear a helmet. For good health wear a condom  **Graphic Text:** Safety  **Bottom Poster Message:** Building a city takes a lot of hard work; preventing AIDS is even more important.  **Text under man:**  Lu Liang  Shanghai AIDS Prevention Spokesperson  **Text in blue area:**  Shanghai CDC  China-Canada AIDS Collaborative Project  Pudong, Xiuhui, Nanhui, Songjiang CDCs  Hotline: 63010910 (Shanghai); 53074900 (Pudong); 64741079 (Xuihui); 68018391 (Nanhui); 37731090 (Songjiang) | **Exhibition Poster (Movable display board, Poster 2):** Man pointing at title and two germs pointing in the lower left corner  **Title:** Do you know about AIDS?  **Paragraph One:**  AIDS is a serious infectious disease that has a very high death rate, presently there is no effective vaccine and cure, but it is totally preventable.  **Paragraph Two:**  When infected with HIV you become an HIV infected person, before they become ill, the person’s appearance is the same as a healthy person, but blood, semen, vaginal secretions, and breast milk all carry the virus, and it can be transmitted to others. |
